# Quality of Japanese Online Information on Causes of Neck Pain: A Biopsychosocial Analysis

**DOI:** 10.7759/cureus.41353

**Published:** 2023-07-04

**Authors:** Yu Kondo, Rumi Ota, Hisaki Fujita, Takahiro Miki, Yuta Watanabe, Tsuneo Takebayashi

**Affiliations:** 1 Rehabilitation, Sapporo Maruyama Orthopedic Hospital, Sapporo, JPN; 2 Rehabilitation, Tokachi Orthopedic Clinic, Obihiro, JPN; 3 Orthopedic, Sapporo Maruyama Orthopedic Hospital, Sapporo, JPN

**Keywords:** online infomation, neck pain, biopsychosocial, non-specific neck pain, online media

## Abstract

Background

While a considerable amount of information on neck pain is available online, the quality and comprehensiveness of this information can vary greatly. Particularly, the representation of the biopsychosocial model - which recognizes neck pain as an interplay of biological, psychological, and social factors - in online information remains unclear. Given the prevalence and accessibility of online health information, it is important to understand its quality and how it may shape individuals' understanding and management of neck pain. Therefore, the objective of this study was to analyze the quality of online Japanese information on the causes of neck pain from a biopsychosocial perspective.

Methodology

A descriptive cross-sectional study was conducted. The search term "neck pain" was utilized on Google's search engine in June 2023, and the websites from the first two pages of the search results were included in the study. Ten advertisement websites were excluded, resulting in 19 websites being analyzed. Each website was evaluated based on the presence of the Health-on-the-Net (HON) code. Biomedical and psychosocial factors present in each website were identified using the biopsychosocial analysis tool. Websites were then categorized as biomedical, limited biopsychosocial, or biopsychosocial based on the number of psychosocial factors they mentioned.

Results

Among the 19 evaluated websites, only one possessed the HON certification, indicating a potential lack of credibility for the remaining sites. Of these websites, a large majority (63.2%) were classified as biomedical, while the remaining (36.8%) were classified as limited biopsychosocial. All the websites included some form of biomedical information on the causes of neck pain, while only seven websites mentioned psychological factors and one website mentioned social factors. The most common biomedical causes of neck pain discussed were cervical muscle strain and radicular pain due to cervical disc prolapse. On the other hand, the limited biopsychosocial websites highlighted perceived stress, depressed mood, and job-related mental stress as psychosocial factors contributing to neck pain.

Conclusions

This analysis revealed that freely accessible Japanese online information on the causes of neck pain, as found through Google, predominantly focuses on the biomedical causes, often neglecting or insufficiently addressing the psychosocial aspects. This finding underscores a gap between the available online resources and the comprehensive understanding promoted by the biopsychosocial model of health. Healthcare professionals need to be proactive in guiding their patients toward reliable, well-rounded resources that acknowledge the crucial role of psychosocial factors in neck pain. Furthermore, developers of online health information must aim to improve the depth and breadth of psychosocial factors discussed, promoting a more holistic understanding of neck pain for the Japanese public.

## Introduction

Neck pain is a common musculoskeletal pain disorder that is recognized as a major health problem in Japan [1]. Neck pain is characterized by chronicity and recurrence and is associated with poor quality of life and activities of daily living among the Japanese general population [2]. As the biopsychosocial model has become increasingly understood, it is clear that there is a complex and interdependent relationship between the biomedical and psychosocial factors related to neck pain [3]. A recent review has shown that a variety of psychosocial factors, such as stress, anxiety, depression, fear of exercise, pain catastrophizing, coping, self-efficacy, low job satisfaction, and high job strain can influence outcomes in patients with neck pain [4]. These psychosocial contributors have critical implications for the management of neck pain [5]. Therefore, patient education based on the biopsychosocial model of the causes of neck pain is an essential therapeutic component in the management of neck pain.

It has been reported that many patients search the Internet to gather health-related information. Yamamoto et al. [6] in a study of access to and awareness of health-related information among Japanese reported that 36.8% of people used the Internet for active collection of health-related information in 2017. Furthermore, 24.1% of respondents reported using search engines such as Google as the most used source of information when gathering health-related information, which was comparable to consulting a doctor (28.9%) or a pharmacist (23.3%). The Internet has become a major source of health-related information, and such information is likely to influence the medical decision-making and healthcare choices of patients with neck pain [7]. Therefore, there is growing interest in studies assessing the quality of information on the Internet [7-9]. Previous studies have shown that online information can change the knowledge of chronic patients and have a positive impact on their attitudes and behaviors, especially regarding physical and mental health problems [10]. For the Internet to become an effective source of patient education regarding neck pain, it is important to understand current online information and analyze the quality of online information about the causes of neck pain from the perspective of the biopsychosocial model. Previous studies have estimated that the quality of health-related information on the Internet for neck pain is low [7,8], and online information on psychosocial factors about the causes of neck pain is inadequate [9].

However, these studies have focused on online English information. This study aims to fill the gap by analyzing the quality of Japanese online information on the causes of neck pain from the perspective of biopsychosocial models.

## Materials and methods

Ethics approval and consent to participate

This research did not involve any human research subjects and only included written texts publicly available on the Internet; therefore, ethics approval or consent to participate was not required. Online information was anonymized and used.

Search engine and strategy

A descriptive cross-sectional study was conducted to analyze online information regarding the causes of neck pain. An online search was conducted using Google’s search engine on June 28, 2023, using the keyword "neck pain." Google was used because it accounts for more than 77% of searches in Japan as of May 2023 and is the most popular search engine compared to other search engines [11]. To minimize the potential influence of previous search history and browsing activities that might be linked to the IP address used, a Virtual Private Network (VPN) was employed. The VPN served to anonymize the IP address, thereby enhancing the objectivity of the search results. Google Chrome was used as the Internet browser, and the search was conducted in incognito mode so that personal search history and browsing sites would not affect preference analysis. The search strategy was designed to be the keywords most likely to be used by the general public when they attempt to find information regarding neck pain [8,9]. The keyword "neck pain" was selected because it is searched more frequently in the Japanese-speaking world than "cervical spondylosis," "cervical disc herniation," and "cervicobrachial syndrome" in Google Trends [12].

Website identification and categorization

In this study, we referred to the website inclusion criteria developed by Neelapala et al. [9], a comprehensive study that evaluated the quality of online information on neck pain from a biopsychosocial perspective. Their criteria for the inclusion and exclusion of websites and the methods for evaluating online information provided a robust framework that we adapted for our study. The inclusion criteria for the websites in our study were as follows: 1) a website describing the causes of neck pain in Japanese and 2) freely accessible to the general public. Websites that met the following criteria were excluded: 1) textbooks, 2) published scientific papers, 3) personal blogs, 4) promotional websites, 5) websites that required a subscription fee to access content, 6) websites that had no information on neck pain, and 7) websites written in languages other than Japanese. As online users tend not to visit websites after the first few pages of search results [13], we chose websites in the first two pages for review. The websites were extracted independently by three authors (YK, RO, and HF). Any disagreements regarding eligibility were resolved through discussion. The information on the website was independently evaluated by three authors (YK, RO, and HF) [14]. Any disagreements regarding the evaluation of information on the website were resolved by discussion. The information on the website was evaluated based on the biopsychosocial analysis tool [9] and the Health-on-the-Net (HON) code [9]. Previously reported checklist guidelines were consulted before evaluation to minimize inter-author variability in the evaluation of website information [15]. Website authorship was categorized as academic, private enterprise, physician, nonphysician, and unspecified.

Quality assessment

To identify the number of websites certified by established benchmarks for quality health information, the presence of the HON code was identified [9]. The HON criteria were developed in 1996 by a Swiss-based nonprofit group in an attempt to improve the quality of Internet-based health information. Previous studies demonstrate that websites using the HON code quality standards are more reliable than those that do not [16,17]. 

Biopsychosocial analysis

A qualitative assessment of online information on the causes of neck pain was performed for biomedical and psychosocial factors based on the biopsychosocial analysis tool developed by Neelapala et al. [9] This checklist was developed after an extensive review of the literature on psychosocial factors involved in neck pain and available information on biomedical factors of neck pain on the website of the International Association for the Study of Pain (IASP) [9]. The checklist includes biomedical factors described by the IASP as causes of neck pain: fracture, infections, neoplasm, metabolic bone disease, arthritis, congenital vertebral anomaly, cervical sprain, torticollis, cervical discogenic pain, cervical zygapophyseal joint pain, cervical muscle strain, cervical trigger point syndrome, alar ligament sprain, cervical segmental dysfunction, radicular pain due to cervical disc prolapse, and traumatic avulsion of the nerve roots. The psychological factors included descriptions of depressed mood, perceived stress, generalized anxiety, coping patterns, self-efficacy, fear of movement, and pain catastrophizing. Social factors included descriptions of social support, job satisfaction, job-related mental stress, and the work and family environment. 

Data analysis

The results of the quality of online information were calculated using descriptive statistics. This study used a similar methodology as the report by Neelapala et al. [9]: websites with only descriptions of biomedical factors were classified as biomedical, websites with fewer than three descriptions of psychosocial factors were classified as limited biopsychosocial, and websites with three or more descriptions of psychosocial factors were classified as biopsychosocial [9].

## Results

Website identification and categorization

Google generated 37,100,000 links using the search term "neck pain." Of the websites up to the first two pages, after eliminating the 10 advertisement websites, 19 independent websites remained and were included in the review (Table 1). Among the authors, 63.2% were physicians, 31.6% were from private enterprises, and 5.2% were from academia.

**Table 1:** Analysis of individual websites (n = 19). HON code, Health-on-the-Net code

	*n* (%)
Biomedical factors	
Fracture	0 (0.0)
Infections	10 (52.6)
Neoplasm	6 (0.0)
Metabolic bone disease	0 (0.0)
Arthritis	7 (36.8)
Congenital vertebral anomaly	0 (0.0)
Cervical sprain	10 (52.6)
Torticollis	2 (10.5)
Cervical discogenic pain	7 (36.8)
Cervical zygapophyseal joint pain	8 (42.1)
Cervical muscle strain	15 (78.9)
Cervical trigger point syndrome	0 (0.0)
Alar ligament sprain	0 (0.0)
Cervical segmental dysfunction	0 (0.0)
Radicular pain due to cervical disc prolapse	15 (78.9)
Traumatic avulsion of nerve roots	0 (0.0)
Psychological factors	
Perceived stress	6 (31.6)
Generalized anxiety	0 (0.0)
Depressed mood	2 (10.5)
Fear of movement	0 (0.0)
Pain catastrophizing	0 (0.0)
Coping patterns	0 (0.0)
Self‐efficacy	0 (0.0)
Social factors	
Job satisfaction	0 (0.0)
Job related mental stress	1 (5.3)
Work and family environment	0 (0.0)
Social support	0 (0.0)
Classification in biopsychosocial analysis tool	
Biomedical	12 (63.9)
Limited biopsychosocial	7 (36.8)
Biopsychosocial	0 (0.0)
HON code	1 (5.3)
Authorship	
Academic	1 (5.2)
Private enterprise	6 (31.6)
Physician	12 (63.2)
Nonphysician	0 (0.0)

Quality assessment

One website had HON certification, and neither HON certificate was displayed on the remaining 18 websites (Table 1).

Biopsychosocial analysis

All 19 websites provided information on the biomedical factors of neck pain. Psychological factors were described by seven websites, and social factors were described by one website. Thus, of the 19 sites, 12 were classified as biomedical (63.2%) and seven as limited biopsychosocial (36.8%) (Table 1). The result of the biopsychosocial analysis tool by the Website authorship category were physicians (biomedical 58.3% and limited biopsychosocial 41.7%), private sector (biomedical 66.7% and limited biopsychosocial 33.3%), and academia (biomedical 100%). The top three most common biomedical factors were cervical muscle strain (78.9%), radicular pain due to cervical disc prolapse (78.9%), cervical sprain (52.6%), and infections (52.6%) (Table 1). The limited biopsychosocial website lists perceived stress, depressed mood, and job-related mental stress as psychosocial factors of neck pain (Table 1).

## Discussion

In this study, we analyzed the quality of online Japanese information on the causes of neck pain from the perspective of the biopsychosocial model. The results showed that Japanese online information on the causes of neck pain, freely accessible via the Google search engine, contains limited or no information on the psychosocial factors of neck pain. These results support the results of previous studies that emphasized the inadequate representation of online information on the psychosocial factors of neck pain and chronic low back pain [9,15].

Our analysis of 19 websites revealed that all provided information on the biomedical factors of neck pain, with 63.2% being classified as strictly biomedical in nature. The most common biomedical explanations given for the cause of neck pain were cervical muscle strain and radicular pain due to cervical disc prolapse, both featured on 78.9% of the sites. These factors certainly bear relevance as nociceptive inputs from the disc and cervical muscles can be an important consideration in some patients with neck pain [18]. However, given the majority of clinical diagnoses of cervical pain are nonspecific, and the fact that there is not always a relationship between pathological anatomical changes in the cervical region and clinical symptoms [19,20], it can be misleading to focus solely on pathoanatomic causes in online information [15]. Indeed, patients with chronic pain often hold a strong belief in the biomedical model that pain is due to tissue damage or structural fragility [21], a misconception that may be reinforced by an overemphasis on biomedical factors in online information. Several conceptual models suggest that such improper beliefs can contribute to the development and persistence of chronic pain. For instance, the fear-avoidance model illustrates how pain perceived as threatening, coupled with catastrophizing thoughts, can trigger pain-related fear and anxiety, leading to a vicious cycle of avoidance behavior, disability, and chronic pain [22]. Therefore, the potential impact of these predominantly biomedical model-focused descriptions currently available online on the beliefs of patients with neck pain warrants further research.

Despite the wealth of literature demonstrating the importance of psychosocial factors in the development and persistence of neck pain [3,4,23], more than half of the websites did not mention these critical psychosocial factors. Even when mentioned, the focus was primarily on perceived stress, depressed mood, and job-related mental stress. Major psychosocial contributors such as pain catastrophizing and fear of movement were largely overlooked. This selective and incomplete portrayal of causes for neck pain might not provide sufficient information for patients seeking a comprehensive understanding of their condition. An intervention such as Pain Neuroscience Education could help fill this information gap [24]. This approach aims at shifting patients' understanding of their pain from a mere tissue injury model to a biopsychosocial perspective, emphasizing neural sensitivity and the biology of pain. By informing patients that pain is regulated by many factors, including physical, psychological, and social spheres [24], we can foster a more holistic understanding. However, for the Internet to serve as an effective source of patient education regarding neck pain, it is imperative that online information on the causes of neck pain is presented within a comprehensive framework that adequately addresses psychosocial factors. This finding underscores the need for further research and efforts to improve the representation of psychosocial factors in online information about neck pain.

To reduce the large gap between freely accessible Japanese online information and currently available evidence regarding the causes of neck pain, it is suggested that healthcare professionals take a more active role in guiding patients toward reliable resources, thereby avoiding misinformation and poor decision making [9]. In our results, the majority of the authors of online information were provided by physicians. However, physicians have been reported to be insufficiently trained in the management of chronic pain conditions and have expressed difficulty in adopting and implementing biopsychosocial approaches to back pain management [25]. Despite physicians providing a higher proportion of biopsychosocial information than other authors, more than half still had limited psychosocial information. Moreover, the orientation towards a biopsychosocial approach may be low among Japanese healthcare professionals [26]. As such, enhancing education and implementing the biopsychosocial care model among Japanese healthcare providers could be a significant step towards improving the depiction of online information regarding the causes of neck pain. 

Furthermore, it is essential to advise patients with neck pain to exercise caution when making medical decisions based on the current online information on the causes of neck pain. Previous reports have highlighted the patient's need for explainable causes of low back pain, with patients with chronic low back pain often adhering to the biomedical model of pain and seeking an organic explanation [25]. Some patients even denied the possibility of psychological factors contributing to back pain [27]. Therefore, to rectify these unhelpful beliefs and optimize the delivery of evidence-based therapy, a public health approach is warranted. 

This study has certain limitations that need to be acknowledged. First and foremost, we utilized biopsychosocial analysis tools from previous studies, fully aware of their inherent limitations. These tools, despite their limitations, were chosen because they offer a comprehensive approach that considers the interplay of biological, psychological, and social factors. This choice aligns well with our objective of analyzing the quality of online information on the causes of neck pain from a biopsychosocial perspective. It is also crucial to acknowledge that as of now, there are no universally accepted tools to measure the quality of such online information, which further justifies our choice of this tool. Second, because of the limited number and types of online information analyzed, there is a limit to the generalizable range for assessing the quality of online information. Online information is very much multimedia (i.e. videos, sites such as YouTube/ Facebook/Twitter), and therefore, the true breadth of the biopsychosocial focus of many websites could be gained through further studies analyzing the many images and video clips displayed. Finally, this study was conducted in June 2023. It is unclear to what extent the same results will be reproduced since the study, owing to the rapid and continuous evolution of technology and information on the Internet. Despite these limitations, the results of this study reveal the need to improve the description of Japanese online information about the causes of neck pain and provide important information for the development of websites based on biopsychosocial models.

## Conclusions

In this study, the quality of online Japanese information on the causes of neck pain was analyzed from the perspective of a biopsychosocial model. The results showed that online information on the causes of neck pain in Japanese, which is freely accessible through the Google search engine, contains limited or no information on the psychosocial factors of neck pain. Consequently, the findings of this study underline the necessity to enhance the portrayal of Japanese online information related to the etiology of neck pain. These insights contribute significantly to the future development of web resources grounded in biopsychosocial models.
